# Method for determination of (-102C>T) single nucleotide polymorphism in the human manganese superoxide dismutase promoter

**DOI:** 10.1186/1471-2156-5-33

**Published:** 2004-12-14

**Authors:** Robert CG Martin, Kalista Hughes, Mark A Doll, Qing Lan, Benjamin D Martini, Jolanta Lissowska, Nathaniel Rothman, David W Hein

**Affiliations:** 1 Departments of Surgery, University of Louisville School of Medicine, Louisville, KY, USA; 2 Pharmacology & Toxicology, University of Louisville School of Medicine, Louisville, KY, USA; 3 James Graham Brown Cancer Center, University of Louisville School of Medicine, Louisville, KY, USA; 4 Division of Cancer Epidemiology and Genetics, National Cancer Institute, NIH, DHHS, Bethesda, MD, USA; 5 Division of Cancer Epidemiology and Prevention, Cancer Center and M. Slodowska-Curie Institute of Oncology, Warsaw, Poland

**Keywords:** MnSOD, Human manganese superoxide dismutase, genotyping

## Abstract

**Background:**

Manganese superoxide dismutase (MnSOD) plays a critical role in the detoxification of mitochondrial reactive oxygen species constituting a major cellular defense mechanism against agents that induce oxidative stress. The *MnSOD *promoter contains an activator protein-2 (AP-2) binding site that modifies transcription of *MnSOD*. Mutations have been identified in the proximal region of the promoter in human tumor cell lines. One of these mutations (-102C>T) has been shown to change the binding pattern of AP-2 leading to a reduction in transcriptional activity. The aim of our study was to develop a method to identify and determine the frequency of this (-102C>T) polymorphism in human tissues.

**Results:**

A new TaqMan allelic discrimination genotype method was successfully applied to genomic DNA samples derived from blood, buccal swabs, snap frozen tissue and paraffin blocks. The polymorphism was shown to be in Hardy-Weinberg Equilibrium in an evaluation of 130 Caucasians from Warsaw, Poland: 44 (33.8%) were heterozygous and 6 (4.6%) were homozygous for -102T.

**Conclusion:**

This report represents the first description of the *MnSOD *-102C>T polymorphism in human subjects by a novel Taqman allelic discrimination assay. This method should enable molecular epidemiological studies to evaluate possible associations of this polymorphism with malignancies and other diseases related to reactive oxygen species.

## Background

Antioxidant enzymes such as superoxide dismutase (SOD) protect cells from oxidative stress. Generation of reactive oxygen species (ROS) has been implicated in the etiology of a diversity of human diseases, including cancer[1], aging[2], atherosclerosis[3] and neurodegenerative diseases[4,5]. Superoxide dismutase catalyzes the dismutation of superoxide radical (O_2_^-^) to H_2_O_2 _and O_2_. Three distinct types of SODs have been identified in human cells: 1) a homodimeric cytosolic CuZnSOD [6], 2) an extracellular homotetrameric glycosylated SOD [7], and 3) a mitochondrial matrix homotetrameric manganese superoxide dismutase (MnSOD) [8].

Numerous reports indicate a relative deficiency of superoxide dismutase catalytic activity, including mitochondrial MnSOD, in many types of solid tumors [9,10]. Interest in this relative deficiency of SOD activity has been greatly increased by observations that over-expression of SOD in tumor cells will suppress cell division in culture and tumor growth in vivo[11]. In addition recent reports have suggested a possible association between decreased SOD activity and malignant phenotype[12]. While the precise reasons for this relationship between tumor cell growth rate and intracellular SOD activity are not known, these findings support the general idea that decreased expression of SOD may promote tumor growth. In fact, as a result of these and other observations, MnSOD is considered a tumor suppressor gene[1].

Further evaluation of MnSOD suggests that it is critically important in maintenance of mitochondrial function. Mice with deficiency of this enzyme exhibit progressive cardiomyopathy, neurodegeneration and perinatal death[13]. These studies went on to confirm that transgenic mice that express human MnSOD in the mitochondria are protected from environmental oxygen-induced lung injury [14] and adriamycin-induced cardiac toxicity[15]. In contrast, disruption of the other two SODs yielded viable mice which were normal in non-stressful conditions [16]. Thus the mitochondrial MnSOD represents a major cellular defense against oxidative stress.

Genetic polymorphism in the *MnSOD *mitochondrial targeting sequence has been associated with risks to various diseases including breast cancer[17,18], lung cancer[19], cardiomyopathy[20] and Parkinson's disease[21].

A reduction of MnSOD activity has been shown to exist in many types of human cancer cells when compared to normal cells [22]. A recent report has also demonstrated the possible association between decreased SOD activity and malignant phenotype[12]. A recent report demonstrated a new mutation L60F, in exon 3 of the mature protein in the Jurkat human T-cell leukemia-derived cell line that reduced MnSOD [12]. Thus, it appears that reduced levels of MnSOD activity in human cancer cells can be associated with coding region mutations that alter protein sequence as well as promoter region mutations that alter gene expression [23].

The human *MnSOD *gene is localized to chromosome 6 (6q25). The *MnSOD *promoter region is characterized by a lack of TATA or CAAT boxes but the presence of a GC rich region containing multiple SP-1 and AP-2 binding sites [24]. Further work identified one cause for the reduced expression of MnSOD in some human tumor lines; the occurrence of three heterozygous mutations in the upstream promoter region of this gene [25]. One of these mutations in the *MnSOD *promoter sequence (MnSOD -102C>T) has been shown to change the binding pattern of AP-2 leading to a reduction in transcriptional activity. However the presence of this polymorphism has not been reported in human tissue.

In this study we developed a TaqMan allelic discrimination assay to reliably genotype DNA from many tissues (i.e. blood, buccal swabs, paraffin blocks, and snap frozen tissue) for the -102C>T polymorphism in the *MnSOD *promoter.

## Results

We confirmed the presence of the -102C>T single nucleotide polymorphism in human subjects and submitted the sequence variant to Genbank[26]. The genotyping success rate with this technique in the Polish Caucasian population was 85%. An evaluation of 130 DNA samples successfully genotyped from Polish Caucasians not known to have cancer, demonstrated 80 (61.5%) were homozygous (-102C), 44 (33.8%) were heterozygous (-102CT) and 6 (4.6%) were homozygous (-102TT). This distribution is consistent with the Hardy-Weinberg Law. The success rate with this technique in an additional American control population was blood (95%), buccal swabs (90%), snap frozen tissue (80%) and paraffin-embedded samples (75%). The success rates were influenced by DNA quality, DNA extraction technique, and the ability to acquire enough DNA from the buccal swab.

## Discussion

Reactive oxygen species in the form of superoxide radicals, hydrogen peroxide, and hydroxy-radicals are formed during incomplete reduction of molecular oxygen during normal respirations. The production of reactive oxygen species remains relatively stable during normal physiologic respirations. A significant increase in the production of reactive oxygen species such as superoxide radicals can be greatly increased as a result of metabolic disorders or more commonly from exposure to toxins such as cigarette smoke, well-cooked meat, urban residency, and excessive alcohol consumption.

Under normal physiologic conditions, superoxide radicals are detoxified by superoxide dismutase. Among the three SODs, MnSOD has been demonstrated to be the only form that has been essential for survival of aerobic life [27]. Inactivation of the *MnSOD *gene in *E. coli *significantly increased mutation frequency and cell death when bacteria were grown under aerobic conditions [28]. This has been further demonstrated in the evaluation in mammals in which the inactivation of *MnSOD *gene has led to detrimental effects. Polymorphisms of the human *MnSOD *gene have been found in the promoter region, the sequence coding for mature protein, and the mitochondrial targeting sequence. Initial evaluation of the five prime flanking regions from human tumor cell lines indicated that there were no major additions or deletions in the five prime flanking regions of the human *MnSOD *gene [29]. However, there were three mutations that were identified in these tumor cell lines: a C to a T at the – 102 position; a C to a G at the – 38 and an insertion of an A in 11 straight Gs at the – 93 position in relation to the transcription initiation site. The significance of these mutations was felt to be important because this region includes multiple binding sites for SP-1 proteins as well as AP-2 binding sites. Further evaluation of these mutations identified that the C to T change at the – 102 position effected the overall transcription of the *MnSOD *gene [30]. This change in transcription may result from an effect on the AP-2 binding site. Although the -102 C to T mutation was reported in human tumor cell lines [25], no evaluation has been assessed in human subjects.

Evaluation of the -102C>T polymorphism is complicated by difficulty in PCR because of the excessive GC rich region in which this polymorphism exists. This location, upstream from the transcription start site was extremely difficult to identify through multiple PCR-restriction fragment length polymorphism (RFLP) assays, which failed to adequately digest at this polymorphism site, and led to multiple false negative results. We found only the highest quality DNA (i.e. blood) was able to be evaluated using a PCR RFLP assay with only 50% genotyping success. This failure to accurately reproduce the PCR-RFLP assay [31], led us to the development of this TaqMan allelic discrimination assay.

The TaqMan allelic discrimination assay provided results that were confirmed by automated DNA sequencing and blind repeat genotyping. Although we did not test it use on DNA from multiple tissues from the same individual, it was successful for DNA samples derived from buccal swabs and paraffin blocks. It has significant advantages over RFLP analysis, allele-specific amplification, allele-specific hybridization, and oligo-nucleotide ligation assay techniques. The reasons for this advantage come from the reduction in labor intensive work up, the lack of need for special handling of radioactive probes, and the ability to modify this technique to evaluate multiple polymorphisms in this gene. In addition as more significant polymorphisms within the MnSOD gene are discovered, this technique will facilitate detection within the MnSOD gene.

The limitations of this technique ultimately come from the quality of DNA that is available and the significant initial expense that is required for a TaqMan assay instrumentation.

## Conclusions

This report represents the first description of the *MnSOD *-102C>T polymorphism in human subjects by a novel Taqman allelic discrimination assay. This method should enable molecular epidemiological studies to evaluate possible associations of this polymorphism with malignancies and other diseases related to reactive oxygen species.

## Methods

### DNA sources

Most DNA samples (130) were isolated from buffy coats of Caucasian controls derived from a population-based case-control study of stomach cancer carried out in Warsaw, Poland as previously described [32]. To test the utility of the method to genotype DNA from various tissue sources, peripheral blood (20 samples), buccal swabs (40 samples), paraffin blocks (40 samples), and snap frozen tissues (15 samples) were collected from research subjects in the USA (Louisville, Kentucky).

DNA extraction from paraffin sections was performed after tissue sections (10 sections, 10 μm thick) were cut from paraffin blocks. Samples were removed from paraffin through a sequential extraction with histaclear, 100% ethanol and acetone, and dried under vacuum. The pellet was incubated overnight with proteinase K (200 μg/ml in 50 mM Tris-HCL pH 8.5, 1 mM EDTA and 0.5% Tween-20) at 55°C. After heating at 100°C for 10 min, digestion was sequentially extracted with phenol, phenol/chloroform and chloroform. DNA was precipitated with the addition of 3X volume 95% ethanol.

### Primer design

**Figure 1:**
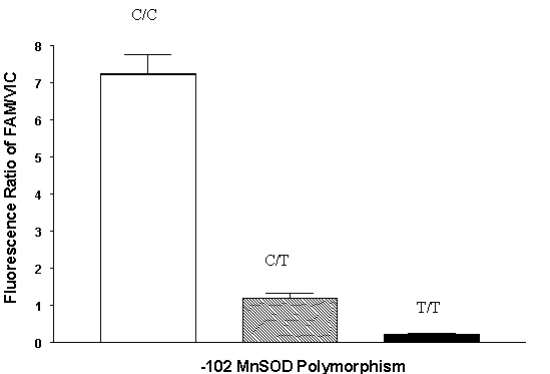
Fluorescence ratios of FAM-labeled/VIC-labeled fluorogenic probes specific for -102C>T polymorphism in *MnSOD*. Each bar represents mean  standard error for determinations in DNA from 3 human subjects. Open bar represents DNA samples homozygous for -102C. Solid bars represent DNA samples homozygous for -102T. Crossed bars represent DNA samples heterozygous for the SNP. The fluorescence ratios differed significantly (p < 0.05) among homozygous and heterozygous genotypes.

SNP-specific polymerase chain reaction (PCR) primers and fluorogenic probes (Table 1) were designed using Primer Express (Version 1.5; Applied Biosystems, Foster City, CA). This technique has been utilized extensively in genotyping other candidate genes with multiple single nucleotide polymorphisms[33,34]. The fluorogenic probes were labeled with a reporter dye (either FAM or VIC) and are specific for one of the two possible bases (-102 C or T) in the *MnSOD *promoter region. A MGB quencher probe was utilized on the 3' end by a linker arm. TaqMan Universal PCR Master Mix (Applied Biosystems) was used to prepare the PCR. The 2X mix was optimized for TaqMan reactions and contained AmpliTaq-Gold DNA polymerase, AmpErase, UNG, dNTPs with UTP and a Passive Reference. Primers, probes and genomic DNA were added to final concentrations of 300 nM, 100 nM, and 0.5–2.5 ng/μl respectively. Controls (no DNA template) were run to ensure there was no amplification of contaminating DNA. Reference control DNA was also utilized to verify the polymorphisms identified. The amplification reactions were carried out in an ABI Prism 7700 Sequence Detection System (Applied Biosystems) with two initial hold steps (50°C for 2 min, followed by 95°C for 10 min) and 50 cycles of a two step PCR (95°C for 15 sec, 60°C for 1 min). The fluorescence intensity of each sample was measured at each temperature change to monitor amplification of the 278 base pair *MnSOD *promoter region. The -102 nucleotide was determined by the fluorescence ratio of the two SNP-specific fluorogenic probes. The fluorescence signal increases when the probe with the exact sequence match binds to the single stranded template DNA and is digested by the 5'-3' exonuclease activity of AmpliTaq-Gold DNA polymerase (Applied Biosystems). Digestion of the probe releases the fluorescent reporter dye (either FAM or VIC) from the quencher dye. As shown in figure 1, the method readily distinguishes between at C or T at -102 in the *MnSOD *promoter region.

**Table 1:** Primers and Fluorgenic Probes for -102C>T *MnSOD *Allelic Discrimination

**Primers**	
-102-Forward Primer (-252 to -234)	5'-gcagacaggcagcgaggtt-3'
-102-Reverse Primer (35 to 19) [287 bp]	5'-ctgaagccgctgccgaa-3'
**Probes**	
-102C-Taqman Probe (-97 to -107)	fam-ccgcgggcccc
-102T-Taqman Probe (-97 to -107)	vic-ccgcgagcccc

Twenty samples with genotypes C/T (4 samples), T/T (3 samples), and C/C (13 samples), some of which were derived from paraffin-embedded tissues, were all confirmed by automated DNA sequencing. These sequence-confirmed samples served as reference standards for the remaining samples. In addition, 10% of the samples were genotyped blind a second time with identical results obtained.

## Author contributions

**RM: **Participated in design of study and manuscript preparation

**KH: **Participated in genotyping samples

**MD: **Participated in design of methods of assay

**QL: **Participated in statistical analysis

**BM: **Participated in design of methods of assay

**JL: **Participated in sample collection

**NR: **Contributed to the study design and the analysis and interpretation of the data

**DH: **Participated in design of study and manuscript preparation
